# A case report of a patient with chronic granulomatous disease complicated by invasive aspergillosis and disseminated *Burkholderia multivorans* infection

**DOI:** 10.3389/fmed.2026.1915139

**Published:** 2026-08-25

**Authors:** Xuanwen Weng, Heng Zhang, Pei Liu, Wei Gao, Huiping Li

**Affiliations:** Department of Pulmonary and Critical Care Medicine, Shenzhen Key Laboratory of Respiratory Diseases, Shenzhen Clinical Research Center for Respiratory Disease, Shenzhen Institute of Respiratory Diseases, Shenzhen People's Hospital (The First Affiliated Hospital, Southern University of Science and Technology, The Second Clinical Medical College, Jinan University), Shenzhen, China

**Keywords:** *Burkholderia multivorans*, chronic granulomatous disease, co-infection, CYBB gene, invasive aspergillosis, missense mutation, X-linked

## Abstract

**Background:**

Chronic granulomatous disease (CGD) is a rare inborn error of immunity characterized by defective phagocyte oxidative burst, leading to recurrent, life-threatening infections with catalase-positive bacteria and fungi. Co-infections with *Aspergillus* and *Burkholderia* species in CGD are exceedingly rare and often fatal due to synergistic pathogenic mechanisms and limited therapeutic options.

**Case presentation:**

We report a fatal case of X-linked CGD in a 14-year-old male with a history of recurrent infections, who presented with severe pneumonia, respiratory failure, and profound growth retardation. Metagenomic next-generation sequencing (mNGS) of bronchoalveolar lavage fluid (BALF) identified *Aspergillus flavus* complex, *Burkholderia multivorans*, and subsequent cultures confirmed disseminated *B. multivorans* infection and invasive aspergillosis. Whole-exome sequencing revealed a novel missense mutation, c.1514T>A (p.Leu505Gln), in the *CYBB* gene, predicted to result in loss of NADPH oxidase function, which is consistent with the severe infectious phenotype observed. Despite aggressive antimicrobial therapy and intensive supportive care, the patient developed refractory septic shock and multiorgan failure, and died on day 14 of hospitalization.

**Conclusions:**

This case underscores the lethal potential of concurrent *Aspergillus* and *Burkholderia* infections in X-linked CGD and highlights the critical importance of early diagnosis, which can be achieved through functional assays such as the DHR test or NBT test, followed by genetic confirmation when available. The novel *CYBB* mutation expands the known genotype-phenotype spectrum of severe X-CGD. Prompt recognition of primary immunodeficiencies in children with recurrent infections caused by typical pathogens is essential to enable timely prophylaxis and curative interventions such as hematopoietic stem cell transplantation before irreversible infectious complications occur.

## 1 Introduction

Chronic granulomatous disease (CGD) is a rare, inherited inborn error of immunity disorder caused by defects in the NADPH oxidase complex (1). This defect impairs the phagocytic respiratory burst, rendering patients' phagocytes incapable of producing sufficient reactive oxygen species to eliminate certain bacterial and fungal pathogens (2). Consequently, individuals with CGD suffer from a heightened susceptibility to severe, recurrent infections and are prone to developing granulomatous inflammation in various organs (3). The spectrum of pathogens in CGD is characteristic, with a predilection for catalase-producing microorganisms (4). The most common microorganisms causing life-threatening infections in patients with CGD are *Staphylococcus aureus, Aspergillus spp., Nocardia spp., Burkholderia spp., Serratia spp.*, and *Salmonella spp*. (1).

CGD is caused by pathogenic variants in one of six genes that encode or permit assembly of the subunits of the phagocyte NADPH oxidase: the X-linked *CYBB* gene (encoding gp91phox, also known as NOX2), and the autosomal recessive genes *CYBA* (encoding p22phox), *NCF1* (encoding p47phox), *NCF2* (encoding p67phox), *NCF4* (encoding p40phox), and *CYBC1* (encoding EROS, an essential chaperone for gp91phox expression) (5). Pathogenic variants in *CYBB, CYBA, NCF1, NCF2*, and *CYBC1* cause classic CGD, characterized by recurrent life-threatening invasive bacterial and fungal infections. In contrast, inherited *NCF4*/p40phox deficiency underlies a distinctive condition that resembles a mild, atypical form of CGD, with patients suffering predominantly from hyperinflammation and peripheral infections, but mainly without the invasive bacterial or fungal infections seen in classic CGD (6). However, rare cases of invasive infections, such as hepatosplenic abscesses, have been reported in patients with p40phox deficiency (7).

While *Aspergillus* and *Burkholderia* infections have been well documented as individual entities in patients with CGD, their co-infection is exceedingly rare in the literature. The underlying synergistic mechanism—potentially involving an exacerbated inflammatory response that leads to irreversible tissue damage and multiorgan failure—remains poorly understood. In the present case, the patient developed concurrent invasive aspergillosis and disseminated *Burkholderia cepacia* infection and ultimately succumbed to refractory septic shock, suggesting that this particular combination may represent one of the most lethal infectious challenges in CGD.

The diagnosis of CGD should begin with functional screening assays—the dihydrorhodamine (DHR) flow cytometric test or the nitroblue tetrazolium (NBT) slide test—both of which can identify defective phagocyte respiratory burst within hours (8, 9). Genetic testing, while essential for confirming the specific gene defect and guiding genetic counseling, is slower and may not be available in resource-limited settings (8). In this case, whole-exome sequencing was performed after the patient's admission; however, earlier functional testing could have facilitated a more timely diagnosis.

In addition, we identified a novel missense mutation, c.1514T>A (p.Leu505Gln), in the *CYBB* gene. This mutation is situated within a conserved functional domain encoding the gp91∧(phox) subunit and results in complete loss of NADPH oxidase activity. This finding not only confirms the diagnosis of X-linked CGD but also contributes to the growing body of evidence on genotype–phenotype correlations: the mutation is closely associated with the patient's disease onset in early childhood, profound growth retardation, and marked susceptibility to both *Aspergillus* and *Burkholderia* species.

By presenting this rare case in detail, we aim to highlight the potentially synergistic lethality of dual opportunistic infections and underscore the clinical relevance of identifying novel *CYBB* mutations in understanding severe X-CGD phenotypes. This report may also serve as a reference for early clinical recognition and precision management of similar high-risk patients.

## 2 Case presentation

### 2.1 Past medical history

The patient was a 14-year-old boy with a history of recurrent infections since early childhood, with an average of 3–4 infectious episodes requiring medical attention per year, including cervical lymphadenitis requiring drainage at age 4 and a liver abscess surgically treated at age 8. He was hospitalized at least three times for infectious episodes prior to this admission. He had never received prophylactic antimicrobial or antifungal therapy (e.g., trimethoprim-sulfamethoxazole, itraconazole) before this hospitalization, nor had he undergone prior immunological evaluation. Six months prior to this admission, he was diagnosed with pneumonia at a local hospital (details unavailable). He had received routine childhood vaccinations according to the national immunization schedule, with no reported adverse events. His parents were healthy and non-consanguineous, with no family history of inborn error of immunity.

### 2.2 Presentation at admission

He presented to our hospital with a 7-day history of productive cough, 3 days of progressive shortness of breath, and a half-hour episode of loss of consciousness. On examination, he was markedly underweight (26 kg, corresponding to the 50th percentile for an 8-year-old) and in respiratory distress. Arterial blood gas analysis (on 61% oxygen via face mask) revealed severe metabolic acidosis with pH 7.219, ${\text{HCO}}_{3}^{-}$ 9.2 mmol/L, base excess −19.0 mmol/L, and respiratory compensation (PaCO_2_ 22.4 mmHg). The PaO_2_ was 133 mmHg, giving a PaO_2_/FiO_2_ ratio of approximately 218, consistent with type I respiratory failure. Serum lactate was markedly elevated at 13.72 mmol/L. Laboratory tests showed leukocytosis (WBC 29.17 × 10^9^/L), mild anemia (Hb 106 g/L), marked thrombocytosis (platelets 634 × 10^9^/L), and elevated inflammatory markers (CRP 41.5 mg/L, PCT 0.21 ng/mL). Additional abnormalities included hyponatremia (Na 119 mmol/L), hypochloremia (Cl 80 mmol/L), and elevated AST (60 U/L), indicating early multiorgan involvement

### 2.3 Admission chest CT

**Figure 1:**
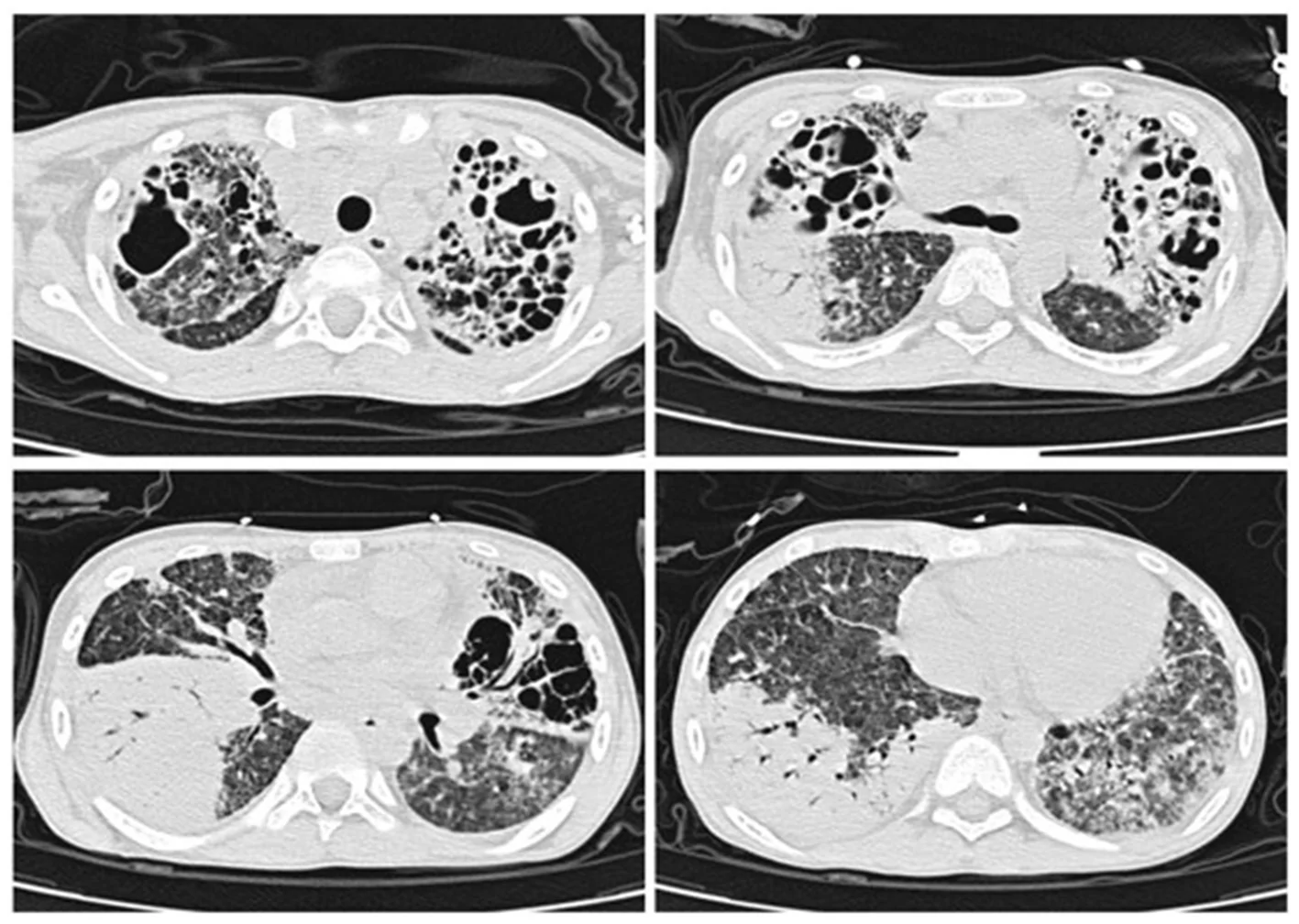
Chest computed tomography (CT) of the patient. Transverse CT images revealed bilateral pulmonary infiltrates, right lower lobe consolidation, cystic lesions in both upper lobes, and mediastinal lymphadenopathy.

Chest computed tomography revealed bilateral pulmonary infiltrates, consolidation in the right lower lobe, cystic lesions in both upper lobes, and mediastinal lymphadenopathy (Figure 1).

### 2.4 Hospital course and diagnosis

*Initial management*: Empirical therapy with doxycycline and meropenem was initiated, along with non-invasive ventilation and supportive care.

*Day 3*: Metagenomic next-generation sequencing (mNGS) of bronchoalveolar lavage fluid (BALF) detected Staphylococcus aureus (13,605 sequence reads), Haemophilus influenzae (99 reads), Burkholderia multivorans (1,346 reads), Candida albicans (109 reads), and Aspergillus flavus complex (3 reads). Based on these findings, the antimicrobial regimen was switched to a combination of meropenem, linezolid, and voriconazole, and doxycycline was discontinued.

*Day 4*: The patient developed worsening tachypnea, tachycardia, and declining oxygen saturation, requiring endotracheal intubation and invasive mechanical ventilation. Owing to severe hypoxemia, prone ventilation was implemented. Bronchoscopy revealed copious purulent secretions.

*Day 10*: Sputum culture grew B. multivorans (Burkholderia multivorans). Antimicrobial susceptibility testing demonstrated susceptibility to meropenem and minocycline; therefore, minocycline was added.

*Day 11*: Blood culture grew B. multivorans.

*Day 12*: Bronchoscopy showed white plaques in the airway. Targeted high-throughput sequencing of BALF was performed again, which detected Pseudomonas aeruginosa and Aspergillus fumigatus.

*Day 14*: The patient died of multiple organ failure.

*Genetic diagnosis*: Whole-exome sequencing revealed a novel missense mutation in the *CYBB* gene, c.1514T>A (p.Leu505Gln). According to ACMG/AMP guidelines, this variant meets PM1 (located in a critical functional domain), PM2 (absent from population databases), PP3 (multiple *in silico* tools predict deleterious effect), and PP4 (patient's phenotype highly specific for CGD), supporting a **Pathogenic** classification. The variant has not been previously reported in ClinVar, HGMD, or gnomAD, confirming its novelty.

Based on the EORTC/MSGERC criteria (10), the patient fulfilled the requirements for probable invasive aspergillosis, given the presence of host factors (X-linked CGD), clinical evidence (CT showing bilateral infiltrates, consolidation, and cystic lesions), and microbiological evidence (BALF mNGS detecting *Aspergillus* species). Histopathological confirmation and serum/BAL galactomannan assays could not be obtained due to the patient's critical condition and rapid deterioration.

**Figure 2:**
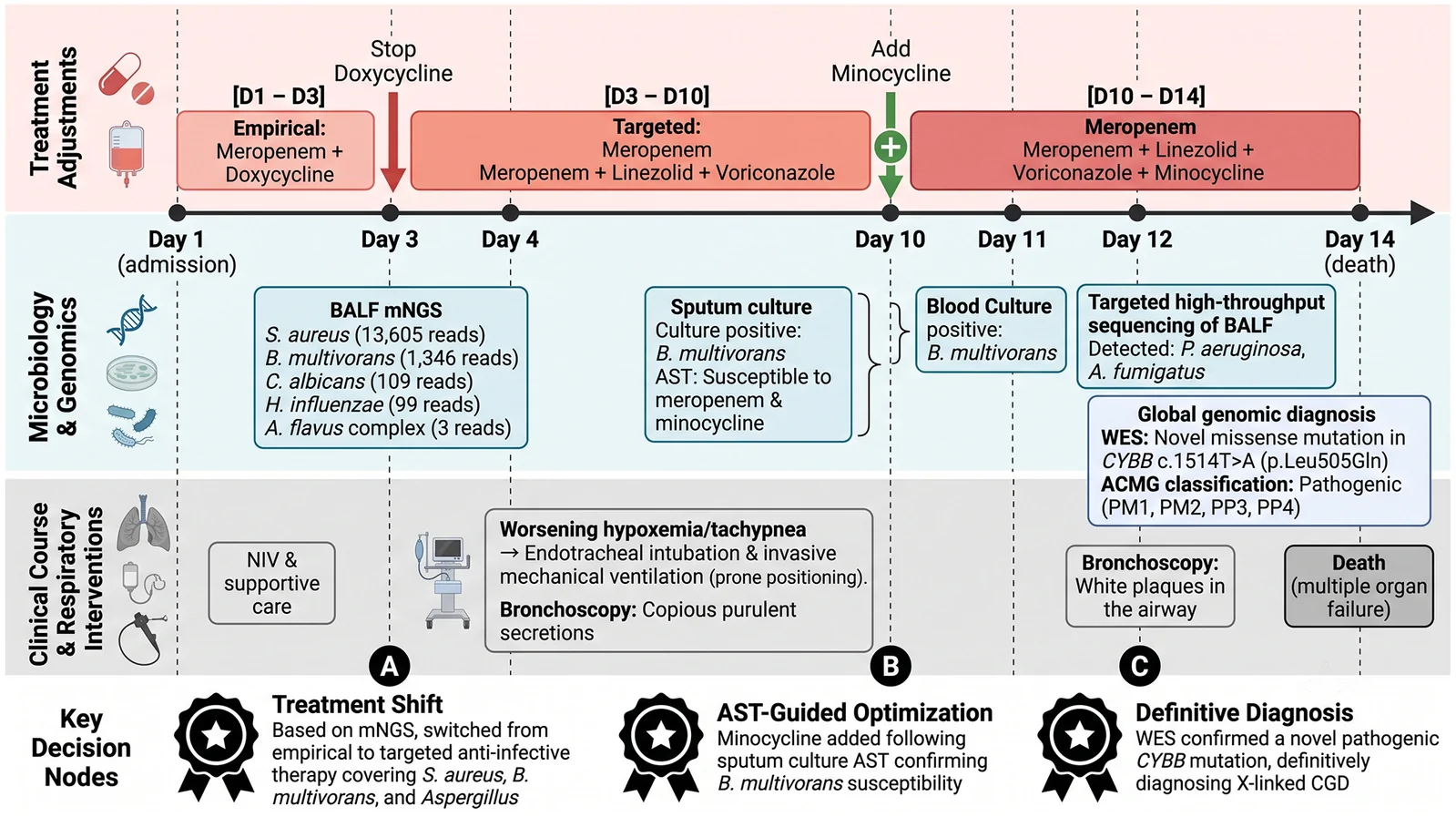
The timeline summarizes key diagnostic and therapeutic milestones from admission to death. On Day 1, empirical antibiotics and noninvasive ventilation were initiated for severe pneumonia and septic shock. On Day 3, BALF mNGS identified multiple pathogens, prompting adjustment to targeted antimicrobial therapy. On Day 4, respiratory deterioration necessitated intubation and prone ventilation. Sputum and blood cultures confirmed disseminated Burkholderia multivorans infection, and the genetic diagnosis of X-linked CGD (CYBB c.1514T>A) was established on Day 10. Despite maximal intensive care, the patient developed refractory septic shock and multiorgan failure, and died on Day 14.

A comprehensive timeline integrating treatment modifications, microbiological results, and clinical events is presented in Figure 2.

### 2.5 Final outcome

Despite maximal intensive care, the patient's respiratory function deteriorated progressively, with refractory hypercapnia and hypoxemia. Inflammatory markers and lactate continued to rise, and septic shock became unresponsive to vasopressors. He died on day 20 of hospitalization due to multiorgan failure.

## 3 Discussion

This case presents the fatal outcome of a 14-year-old boy with chronic granulomatous disease, whose clinical course was characterized by the concurrent presence of invasive fungal infection and disseminated *Burkholderia multivorans* bacteremia, and the combination of these two pathogens is suggested to have contributed to the fatal outcome in the context of underlying CGD. The diagnosis of X-linked CGD, confirmed by the identification of a novel pathogenic variant in the *CYBB* gene, is central to understanding the profound and ultimately intractable nature of his immunodeficiency. To provide a structured analysis of this complex case, the Discussion is organized into three sections: (1) the synergistic lethal mechanism of co-infection; (2) the correlation between the novel genotype and severe clinical phenotype; and (3) the clinical significance of growth retardation as a marker of disease severity. We acknowledge that while structural homology modeling provides valuable *in silico* evidence, it does not replace functional assays. The conclusions regarding the biological impact of the novel *CYBB* variant are therefore expressed with appropriate caution, and functional studies in future cases are warranted.

### 3.1 The synergistic lethal mechanism of co-infection

The patient's infection profile represents a classic yet devastating convergence of the two most feared pathogens in CGD (1, 4). The simultaneous occurrence of invasive aspergillosis and disseminated *B. multivorans* infection likely constituted a particularly challenging clinical scenario that overwhelmed therapeutic interventions through complex pathogen-pathogen and pathogen-host interactions.

#### 3.1.1 *Burkholderia multivorans*: intracellular persistence and antibiotic resistance

Members of the *B. cepacia* complex, particularly *B. multivorans*, are notorious for causing severe, often fatal infections in CGD due to multiple virulence factors (1, 4). Their intrinsic resistance to many β-lactams and aminoglycosides complicates empirical therapy (11), but more critically, their ability to survive intracellularly within dysfunctional CGD phagocytes facilitates unchecked hematogenous dissemination. In this case, positive blood cultures on Day 11 confirmed metastatic spread, while the persistence of elevated inflammatory markers (CRP 41.5 mg/L on admission) and refractory hyperlactatemia (13.72 mmol/L) despite targeted therapy suggested ongoing intracellular replication and tissue hypoperfusion. The fundamental phagocytic defect rendered antibiotic treatment largely ineffective—antimicrobial agents could not penetrate the intracellular niche, and the host immune system lacked the respiratory burst required to clear the pathogen even when antibiotics were present (2). This phagocytic incapacity directly contributed to refractory septic shock.

#### 3.1.2 *Aspergillus species*: tissue invasion and inflammatory dysregulation

Invasive aspergillosis remains the leading cause of mortality in CGD (1, 4). The dynamic shift from *A. flavus* complex (detected by mNGS on Day 3) to *A. fumigatus* (identified on Day 10) illustrates the progressive and polymicrobial nature of fungal invasion. Voriconazole, while the first-line therapy, cannot compensate for the complete absence of neutrophil-mediated hyphal damage. Recent studies indicate that NADPH oxidase functions as a critical regulator of pulmonary antifungal immunity, and its absence in CGD leads to dysregulated neutrophil accumulation, hyperinflammation, and paradoxically ineffective pathogen clearance (12)—a phenomenon clearly demonstrated by the diffuse airway leukoplakia and copious purulent secretions observed during bronchoscopy in this case.

It is noteworthy that initial BALF mNGS detected only 3 reads of *Aspergillus flavus* complex, while a subsequent test identified *Aspergillus fumigatus*. This low abundance in mNGS should not be dismissed, as fungal DNA extraction efficiency from BALF is inherently low due to high human DNA background and the thick fungal cell wall (13). Although the optimal threshold for distinguishing *Aspergillus* infection from colonization by BALF mNGS has been reported as 23 reads (14), the detection threshold for fungal pathogens is generally set at SMRN ≥ 3 (15). More importantly, in the context of an immunocompromised host with compatible radiographic findings and progressive clinical deterioration, even low reads of *Aspergillus* are clinically meaningful (16). The temporal shift from *A. flavus* to *A. fumigatus* may reflect polymicrobial co-infection or changes in relative fungal burden over time, but the consistent detection of *Aspergillus* species across two independent tests supports a unified diagnosis of invasive aspergillosis and argues against contamination. Furthermore, mNGS results should be interpreted in conjunction with conventional microbiological tests; in this case, while fungal cultures remained negative (possibly due to prior antifungal therapy), mNGS provided critical microbiological evidence that traditional methods could not offer. Voriconazole remains the standard therapy for both *A. flavus* and *A. fumigatus*, and thus the species shift did not alter clinical management (17).

#### 3.1.3 The vicious cycle of polymicrobial synergy and treatment failure

We hypothesize that the co-infection may have exerted a synergistic effect that contributed to the treatment failure. Bacterial sepsis induced systemic inflammatory response syndrome (SIRS), compromising vascular integrity and tissue oxygenation, which in turn facilitated fungal angioinvasion. Conversely, fungal infection caused cavitary lung lesions (evident as cystic changes on initial CT) that served as reservoirs for bacterial persistence and biofilm formation, preventing effective antibiotic penetration. The combined pathogen burden drove an overwhelming cytokine storm, leading to multi-organ dysfunction, as reflected by the initial lactate of 13.72 mmol/L and progressive metabolic decompensation (pH 7.219, ${\text{HCO}}_{3}^{-}$ 9.2 mmol/L). The inability to control either infection exacerbated the other, creating a vicious cycle where antimicrobial therapy, however aggressive, could not achieve microbiological eradication in the context of complete phagocytic incompetence. This polymicrobial synergy represents one of the most formidable challenges in managing advanced CGD and illustrates why, in such a profound immunocompromised state, even maximal intensive care may be insufficient once disseminated infection is established (18).

#### 3.1.4 Therapeutic decision-making in the context of CGD

The treatment modifications in this case were guided by a combination of microbiological findings, antimicrobial susceptibility results, and the patient's clinical trajectory. The initial empirical regimen of meropenem and doxycycline was chosen to cover both community-acquired pathogens and *Burkholderia* species, given the patient's severe pneumonia and septic shock. Upon receipt of mNGS results on Day 3, the regimen was adjusted to meropenem, linezolid (for the copious *S. aureus* detected, 13,605 reads), and voriconazole, while doxycycline was discontinued. On Day 11, positive blood cultures with AST-guided susceptibility to meropenem and minocycline prompted the addition of minocycline. This triple regimen (meropenem + minocycline + voriconazole) was consistent with current recommendations for *Burkholderia cepacia* complex (BCC) infections in CGD, which emphasize aggressive combination therapy guided by AST, as BCC species exhibit intrinsic multidrug resistance (19).

Regarding adjunctive immunomodulatory therapies, recombinant interferon-gamma (IFN-γ) is the only FDA-approved biologic agent for reducing the frequency and severity of serious infections in CGD. A recent systematic review and meta-analysis confirmed that IFN-γ significantly reduces the risk of serious infections in CGD patients (20). However, in this case, genetic diagnosis was not established until Day 10 of hospitalization, when the patient was already in refractory septic shock and multiorgan dysfunction, precluding any meaningful opportunity to initiate IFN-γ prophylaxis.

Corticosteroids are used in CGD primarily for managing granulomatous complications, such as inflammatory bowel disease and genitourinary tract obstruction (20). In recent years, corticosteroids have also been explored as adjunctive therapy for refractory infections in CGD, with several case reports describing successful outcomes when combined with antimicrobial agents (21). However, their use in the setting of active invasive fungal infection remains controversial, as corticosteroids may mask or promote fungal dissemination, particularly *Aspergillus* (21). Given that our patient had concurrent invasive aspergillosis and was hemodynamically unstable, we judged the potential risks of corticosteroid therapy to outweigh any possible benefits, and therefore did not administer corticosteroids.

Notably, *Burkholderia cepacia* complex infection alone can trigger a severe systemic inflammatory response syndrome (SIRS) or even hemophagocytic lymphohistiocytosis (HLH)-like phenotype in CGD patients. Several case reports have demonstrated that immunosuppressive therapy—including corticosteroids, intravenous immunoglobulin, and ciclosporin—may be beneficial in CGD patients with Burkholderia infection, even in the presence of concurrent *Aspergillus* infection (22–24). For instance, Okano et al. (25) reported a 14-year-old X-linked CGD patient with invasive pulmonary aspergillosis and *B. cepacia* co-infection who showed dramatic improvement following methylprednisolone pulse therapy. Similarly, Kaygusuz et al. (22) described a 36-year-old CGD patient with *Burkholderia multivorans/cepacia* complex and *A. niger* co-infection who responded well to high-dose corticosteroids.

In the present case, high-dose corticosteroid therapy was considered but ultimately not administered for the following reasons. First, the patient had active invasive aspergillosis, and corticosteroids may mask or promote fungal dissemination, particularly *Aspergillus*. Second, the patient was hemodynamically unstable upon admission, requiring high-dose vasopressor support. Third, the genetic diagnosis of CGD was not established until Day 10 of hospitalization, by which time the patient had already developed multiorgan dysfunction, precluding any meaningful window for immunomodulatory intervention. These considerations highlight the delicate balance between infection control and inflammation regulation in managing advanced CGD with polymicrobial infections.”

Allogeneic hematopoietic stem cell transplantation (HSCT) is the only curative treatment for CGD and should be evaluated early if an HLA-matched donor is available (26). Current guidelines recommend that HSCT be considered promptly following diagnosis, ideally before the establishment of irreversible infections (26). In this case, the patient was diagnosed with X-CGD only at a terminal stage, when multiorgan failure had already supervened, precluding any opportunity for HSCT evaluation. This tragic outcome reinforces that early genetic diagnosis, coupled with timely HSCT assessment, is essential to alter the natural history of severe X-linked CGD.

It is important to emphasize that the diagnosis of CGD should ideally be established by functional assays rather than relying solely on genetic testing. DHR flow cytometry is the preferred screening method in well-equipped centers, while the NBT slide test remains a valuable alternative in resource-limited settings. Genetic testing, although essential for identifying the specific gene defect and determining inheritance patterns, is not a prerequisite for initiating antimicrobial prophylaxis and infection control measures (8, 9).

To contextualize this case, we reviewed the literature for previously reported cases of CGD with concurrent *Aspergillus* and *Burkholderia* infection. Two additional cases were identified. Table 1 summarizes the clinical and genetic features of all reported cases, including the present one.

**Table 1:** Reported cases of CGD co-infected with *Aspergillus* and *Burkholderia*.

Case	Age/sex	Gene defect	*Aspergillus* species	*Burkholderia* species	HLH/hyperinflammation	Treatment	Outcome
Kaygusuz et al. (22)	36 years/M	*NCF1* (p47phox deficiency)	*A. niger*	*B. multivorans/cepacia* complex	Yes (HLH)	Antibiotics + high-dose corticosteroids + IFN-γ	Improved, planned HSCT
Xu et al. (35)	8 months/M	*CYBB* (c.1234delG)	*Aspergillus spp*.	*B. cepacia*	Not reported	Antimicrobial therapy	Not reported
Present case	14 years/M	*CYBB* (c.1514T>A, p.Leu505Gln)	*A. flavus*→*A. fumigatus*	*B. multivorans*	Yes (SIRS/septic shock)	Antibiotics + antifungals	Died

HLH, hemophagocytic lymphohistiocytosis; HSCT, hematopoietic stem cell transplantation; IFN-γ, interferon-gamma; SIRS, systemic inflammatory response syndrome.

### 3.2 Genotype-phenotype correlation: predicted functional impact of the novel c.1514T>A mutation

The identification of the *CYBB* c.1514T>A (p.Leu505Gln) variant not only confirms the diagnosis but is also suggestive of a severe clinical phenotype, which is in line with the known genotype-phenotype relationships in X-linked CGD, although direct functional confirmation remains unavailable. X-linked CGD, resulting from mutations in the *CYBB* gene encoding the gp91phox subunit, is typically more severe than autosomal recessive forms, and specific mutations within critical domains correlate with distinct clinical outcomes (27, 28).

#### 3.2.1 Structural and functional prediction of the novel mutation

The c.1514T>A mutation results in a leucine-to-glutamine substitution at position 505 of the gp91phox protein. Leucine 505 is located within the highly conserved NADPH-binding domain, and structural homology modeling predicts that this residue resides at the interface between gp91phox and the p22phox subunit. The substitution of a hydrophobic leucine with a polar glutamine is predicted to disrupt domain stability and electron-transfer function, although direct functional validation could not be performed in this acutely deteriorating patient. Nevertheless, the structural prediction, together with the patient's typical clinical phenotype and absence of other pathogenic variants, is consistent with the pathogenic role of this novel mutation as suggested by the ACMG classification and the patient's clinical presentation. A literature review reveals no previous reports of this specific mutation, confirming its novelty. However, mutations at adjacent residues (e.g., p.Leu502Pro, p.Leu508Arg) have been associated with severe X-linked CGD phenotypes and complete enzyme deficiency, providing indirect support for the potential pathogenicity of our finding (27).

We have systematically evaluated this novel variant according to the ACMG/AMP guidelines (28). The variant meets PM1 (located within the NADPH-binding domain, a highly conserved functional domain of gp91phox), PM2 (absent from gnomAD and other population databases), PP3 (multiple *in silico* tools predict deleterious effect), and PP4 (patient's phenotype is highly specific for X-CGD). Based on these combined evidences, the variant is classified as Pathogenic. Due to the parents' refusal of genetic testing, segregation analysis could not be performed, which we acknowledge as a limitation.

#### 3.2.2 Genotype-specific phenotypic manifestations

The predicted loss of NADPH oxidase function is consistent with the patient's severe disease trajectory, although this correlation remains to be confirmed by functional studies. Compared with milder forms such as NCF4/p40phox deficiency, which may present with residual oxidase activity and atypical features, *CYBB* mutations at critical residues like Leu505 typically result in total failure of the respiratory burst (27). If confirmed by future functional studies, this predicted molecular severity would be consistent with the clinical features observed in this patient, including: (1) early childhood onset...; (2) profound susceptibility to catalase-positive organisms...; and (3) poor response to standard prophylaxis and aggressive antimicrobial therapy. The patient's history aligns with established genotype-phenotype correlations in X-linked CGD, where complete deficiency predicts earlier presentation, more frequent hospitalizations, and higher mortality (28, 29).

#### 3.2.3 Therapeutic implications of severe X-linked CGD

The severity of the phenotype underscores the necessity of rigorous, lifelong antimicrobial prophylaxis (trimethoprim-sulfamethoxazole and itraconazole) from early childhood (28). More importantly, it strongly favors early evaluation for allogeneic hematopoietic stem cell transplantation (HSCT), ideally performed before irreversible infections become established. Retrospective data demonstrate that elective HSCT achieves 92% engraftment and 82% long-term survival (29), outcomes that contrast sharply with the dismal prognosis of advanced disseminated infection. Emerging therapies, including lentiviral gene therapy, have shown promise in early-phase trials (20, 30–32), but their efficacy is contingent on pre-treatment infection status. This case tragically illustrates that delayed genetic diagnosis precludes access to these curative interventions.

### 3.3 Clinical significance of growth retardation

The profound growth failure observed in this patient—manifesting as a 14-year-old with the weight of an average 8-year-old and complete absence of pubertal development—represents a critical yet often underappreciated marker of disease severity in CGD.

#### 3.3.1 Growth retardation as a biomarker of disease burden

This extreme phenotype is not coincidental but a direct consequence of sustained disease activity. According to parental recall, the patient's growth trajectory showed progressive deviation from age-appropriate percentiles beginning around school age (6 years), with weight falling to approximately 26 kg (*Z*-score ~-3.0, equivalent to the 50th percentile for an 8-year-old) and height below the 3rd percentile (approximately 148 cm) by the time of admission. Complete absence of pubertal development was noted at age 14. The cumulative impact of recurrent, life-threatening infections creates a chronic hypermetabolic and catabolic state, diverting energy and nutrients from anabolic growth processes (33). Additionally, chronic inflammation-induced anorexia and malabsorption further compromise nutritional status. In this case, the complete lack of pubertal development indicates that the hypothalamic-pituitary-gonadal axis was suppressed due to the significant metabolic investment required for survival taking precedence over sexual maturation. Such growth parameters should alert clinicians to the possibility of an underlying inborn error of immunity, particularly when coupled with infections by characteristic CGD pathogens (33, 34).

### 3.4 Study limitations

We acknowledge several limitations in this case report. First, due to the patient's critical condition at presentation (refractory septic shock and respiratory failure requiring mechanical ventilation) and subsequent rapid deterioration, we were unable to perform functional assays such as DHR oxidation test, NBT test, or ROS production measurements. Second, lymphocyte subset analysis and immunoglobulin quantification could not be completed before the patient's death. Third, segregation analysis could not be performed as the parents declined genetic testing. These limitations preclude a complete functional characterization of the novel *CYBB* variant. Nevertheless, the diagnosis of X-linked CGD is robustly supported by the identification of a pathogenic variant in *CYBB* (ACMG classification: Pathogenic), the patient's typical clinical phenotype (early-onset recurrent infections, profound growth retardation), and the characteristic pathogen spectrum (*Aspergillus* and *Burkholderia multivorans*).

In addition to the above case-specific limitations, we acknowledge several broader methodological limitations inherent to this case report. First, the structural predictions and ACMG-based classification, while supportive, cannot substitute for direct enzymatic confirmation. Second, the structural homology modeling presented in this report provides *in silico* evidence but does not constitute experimental proof of protein dysfunction. Third, although mNGS offered critical microbiological insights, it has inherent limitations, including the inability to distinguish viable from non-viable organisms, colonization from true infection, and the potential for environmental contamination; accordingly, mNGS results were interpreted in conjunction with clinical, radiographic, and conventional microbiological findings. Fourth, and most importantly, as a single observational case report, this study cannot establish causal relationships between the genetic variant, the immunological defect, and the clinical outcomes; all mechanistic interpretations should be regarded as hypotheses supported by previous literature rather than definitive conclusions derived from the current patient. These limitations underscore the importance of future functional studies and larger cohort investigations to further characterize the pathogenicity of this novel variant and the clinical features of X-CGD.

## 4 Conclusion and clinical lessons

This case highlights three key lessons. First, primary immunodeficiencies such as CGD should be considered in any child with recurrent or severe infections caused by characteristic pathogens, and genetic confirmation should be pursued early (4, 33, 34). Second, once disseminated infections—particularly the combination of invasive mold and *Burkholderia*—are established in CGD, even aggressive antimicrobial therapy and maximal supportive care may fail. Third, the cornerstone of managing severe X-linked CGD lies in early genetic diagnosis, timely prophylaxis, and prompt HSCT evaluation, as the prognosis of advanced disseminated disease remains exceedingly poor (28, 29).

## Data Availability

All raw clinical data involved in this case report are protected for patient privacy in accordance with ethical requirements and cannot be publicly deposited in open repositories. All relevant analysis results are fully presented within the manuscript figures and text. We confirm that the anonymized raw data can be reasonably shared upon qualified academic request by contacting the corresponding author Li Huiping ( huipingli2154@163.com) after formal ethical approval application.
